# IBRtools: An R package for calculating integrated biomarker indexes

**DOI:** 10.1002/ece3.10864

**Published:** 2024-02-01

**Authors:** Anna Carolina Resende, Diego Mauro Carneiro Pereira

**Affiliations:** ^1^ Marine Ecology and Ecosystem Modelling Lab, School of Biological Sciences Victoria University of Wellington Wellington New Zealand; ^2^ Carl Peter von Dietrich Glycobiology Laboratory, Biochemistry Department Federal University of São Paulo São Paulo Brazil

**Keywords:** ecophysiology, IBR, IBRv2, radar chart

## Abstract

Multibiomarker studies are useful to evaluate the early warning signs of environmental degradation, and their unified responses are often assessed through two common indexes, Integrated Biomarker Response (IBR) and Integrated Biological Responses version 2 (IBRv2). The R package IBRtools allows users to calculate both IBR and IBRv2 while simultaneously incorporating all the biomarkers under evaluation. The package includes functions for calculating the indexes IBR and IBRv2 and obtaining their standardized values, as well a function for radar chart creation and three example datasets. Here we describe the main algorithms involved in IBR and IBRv2 calculations, a description of the novel package and illustrate a workflow using data examples available on the package to guide the user on how to accurately acquire the values for either the IBR index or the IBRv2 index. The IBRtools package provides a user‐friendly platform for R users to obtain IBR index and IBRv2 values, making it straightforward even for large datasets.

## Graphical abstract

The R package IBRtools allows users to calculate both IBR and IBRv2 with all accessed biomarkers at once and generate a radar chart for visualization, helping with accuracy and unifying multibiomarker studies.

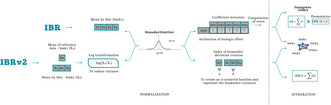

## 1 INTRODUCTION

Evaluating the relationship between stressors and its subsequent biological effects in organisms has been receiving great emphasis (Alfonso et al., 2021; Birnie‐Gauvin et al., 2017; Damiens et al., 2007; Nicolaides et al., 2015; Zimmer et al., 2022). Stress is defined as an external event or condition that generates negative effects in organisms, which can be either abiotic (e.g., variations in pH, temperature, contaminants, and pollution) or biotic (e.g., invasive or predator species; Akbarzadeh et al., 2018; Collier et al., 2017; Petitjean et al., 2019; Schinegger et al., 2012). Environmental stress usually causes physiological responses to metabolic level, where the metabolic rate of organisms is affected and biochemical adjustments are made to ensure homeostasis (Lermen et al., 2004; Przepiura et al., 2019; Resende et al., 2022). With the objective of improving environmental biomonitoring, ecological quality, and risk assessment programs, creating potential lines of evidence to establish cause–effect relationships, many biomarkers have been developed (Colin et al., 2016; Martinez‐Haro et al., 2015; Yancheva, 2016). Biomarkers can be defined as molecular, cellular, histological, physiological, or behavioral observations that predict organisms' imbalance (Colin et al., 2016). These biomarkers are often used as early warning signals before severe environmental degradation occurs (Serafim et al., 2012; Vieira et al., 2014; Xu et al., 2020).

Multibiomarker studies often face the challenge of going beyond an individual interpretation of biomarkers and assessing the global response of the combination of multiple biomarkers (Devin et al., 2017). Given the complexity of multiple biomarker scales and measurements, the need to integrate and unify the results of multiple biomarker studies in a concise way arose. In that context, different data ordinalization methods were created, such as the Health Assessment Index, (Adams et al., 1993), the Biomarker Response Index (Hagger et al., 2006), the Rank Sum Biomarker Index (RSI) (Blaise et al., 2002), the Class Sum Biomarker Index (Chèvre et al., 2003), and the Multimarker Pollution Index (Aarab et al., 2004; J. F. Narbonne, 1999). Thereafter, other integrating methods were created based on data standardization. Beliaeff and Burgeot (2002) proposed the Integrated Biomarker Response (IBR) index, later improved by Devin et al. (2014). The IBR index integrates multibiomarker responses by scaling their values to a common mean, which simplifies its interpretation in biomonitoring software (Beliaeff & Burgeot, 2002; Devin et al., 2014). Later, Devin et al. (2014) updated and optimized this index to avoid misuse. Another type of unifying index has been proposed by Sanchez et al. (2012), called the Integrated Biological Responses version 2 (IBRv2) index. The IBRv2 index integrates multibiomarker responses by comparing their log transformed values to a mean reference data and was created to correct the weak points of the original IBR calculation (Sanchez et al., 2012). Associated with both indexes, a graphic visualization was proposed in the form of radar chart or star plot, in which the standardized biomarker values for each index can be plotted and thereafter their combined responses can be assessed.

The IBR and IBRv2 indexes constitute of popular tools to assess the susceptibility of organisms to ecological risks using multiple biomarker responses and are commonly used as analytical methods to evaluate biomarker responses in many types of organisms, such as fish (Pereira et al., 2022; Serafim et al., 2012; Vieira et al., 2014), mussels (Damiens et al., 2007), annelids (Meng et al., 2016), and plants (Xu et al., 2020).

Despite the importance and common use of the IBR and IBRv2 indexes, there was not yet an open‐source package in R that allowed for the calculation of each index. Researchers have used Excel sheets or web interfaces such as CalIBRi (Devin et al., 2023) to calculate IBR indexes. However, a unified framework in which IBR and IBRv2 can be calculated accurately and in a fast environment is now provided in the novel IBRtools R package. By providing this package in R, we allow for researchers to input their entire biomarker dataset and perform either one of the IBR index analyses while taking advantage of the graphics capabilities also available in the package.

## 2 METHODS

In this section, we describe the six functions available in the IBRtools package (Table 1) and explain how each index and their standardized values are calculated. The graphic visualization is generated using the fmsb package as a base for the IBR and IBRv2 radar charts (Nakazawa, 2023).

**TABLE 1:** List of functions in the IBRtools package.

Functionality	Description
IBR	
*ibr_index*	Calculates the IBR index per site, its mean, and standard deviation
*ibr_std*	Calculates standardized value for each biomarker per site
*ibr_chart*	Generates a radar chart with the standardized values of biomarkers
IBRv2	
*ibrv2_index*	Calculates the IBRv2 index in comparison to the reference site
*ibrv2_bdi*	Calculates standardized values for each biomarker in comparison with the reference site
*ibrv2_chart*	Generates a radar chart with the standardized values of biomarkers

### 2.1 IBR index

#### 2.1.1 Description

**FIGURE 1:**
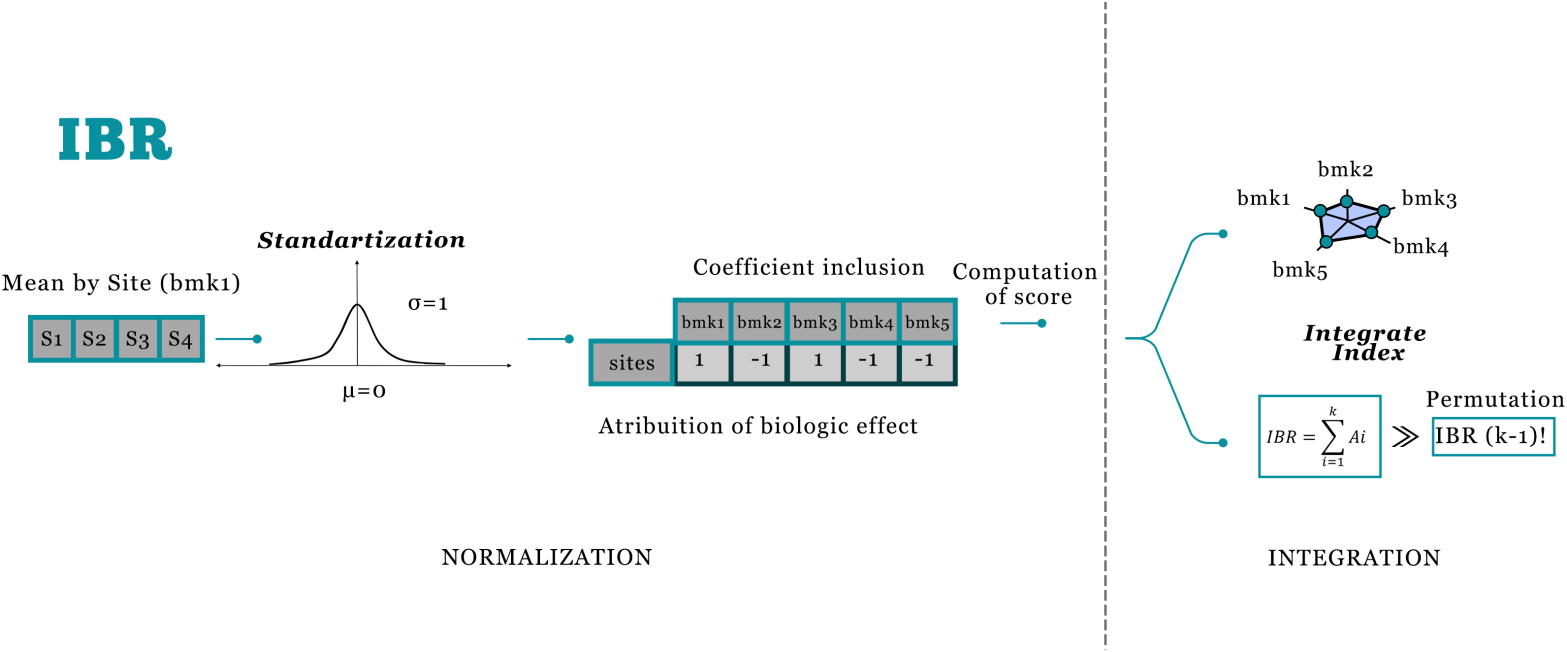
Conceptual flowchart of the integrated biomarker index calculation (IBR). Five hypothetical biomarkers (Bmk1, Bmk2, Bmk3, Bmk4, and Bmk5) are measured in a model organism at four different sites (S1, S2, S3, and S4). Continuous biomarker responses are transformed standardized, where the mean and standard deviation for each site are calculated with all possible circular permutations of k biomarkers for each IBR value. The IBR is calculated as the total area shown by the radar diagram.

The calculation of the IBR proposed by Beliaeff and Burgeot (2002), modified by Devin et al. (2014), presents the steps seen in Figure 1.

**FIGURE 2:**
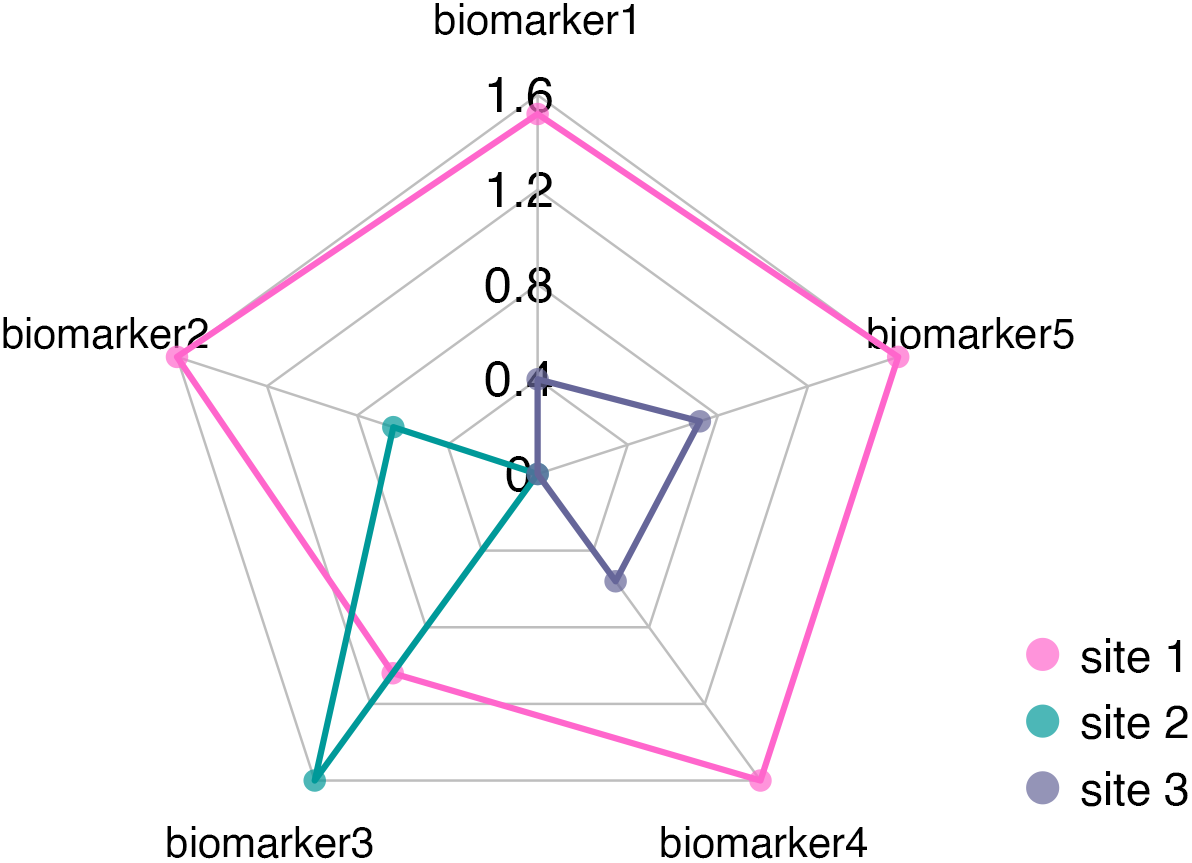
Radar chart for each site obtained through the standardized values with the *enzact* dataset. Five different biomarkers were evaluated and are represented with points on each axis of the graph. The different colors represent the different sites analyzed and the lines set out the area that corresponds to the IBR value for each site.

Figure 2 shows the radar chart performed as a graphic representation of the IBR index from the example dataset included in the package (*enzact*) (Table S1). The area of the radar chart corresponds to the value of IBR (generated with *ibr_index* function) and the value of each enzyme is their standardized form (generated with *ibr_std* function). The IBR analysis will be demonstrated below. The standardized value used to generate the radar chart can be seen in Table S5.

To perform the analysis, three to nine biomarkers with at least three values other than NA must be selected. If the coefficient data frame is not provided, the value 1 will be considered for every biomarker to run the analysis. See the supplementary material for an example of how the coefficient should be arranged (Table S2).

#### 2.1.2 Example of usage



*## install and load package*
install.packages('IBRtools')
library(IBRtools)
*## data load*
data(enzact)
## use of ibr_std function and save output
ibr_std(enzact) ‐〉 ibrstd
## use of ibr_index function and save output
ibr_index(ibrstd) ‐〉 ibrout
*### access each item on the list*
ibrout$IBR_total
ibrout$IBR_mean_sd
*## use of ibr_chart function and save output*
pdf("my_ibrchart.pdf")
ibr_chart(ibrstd)
dev.off()




### 2.2 IBRv2 index

#### 2.2.1 Description

**FIGURE 3:**
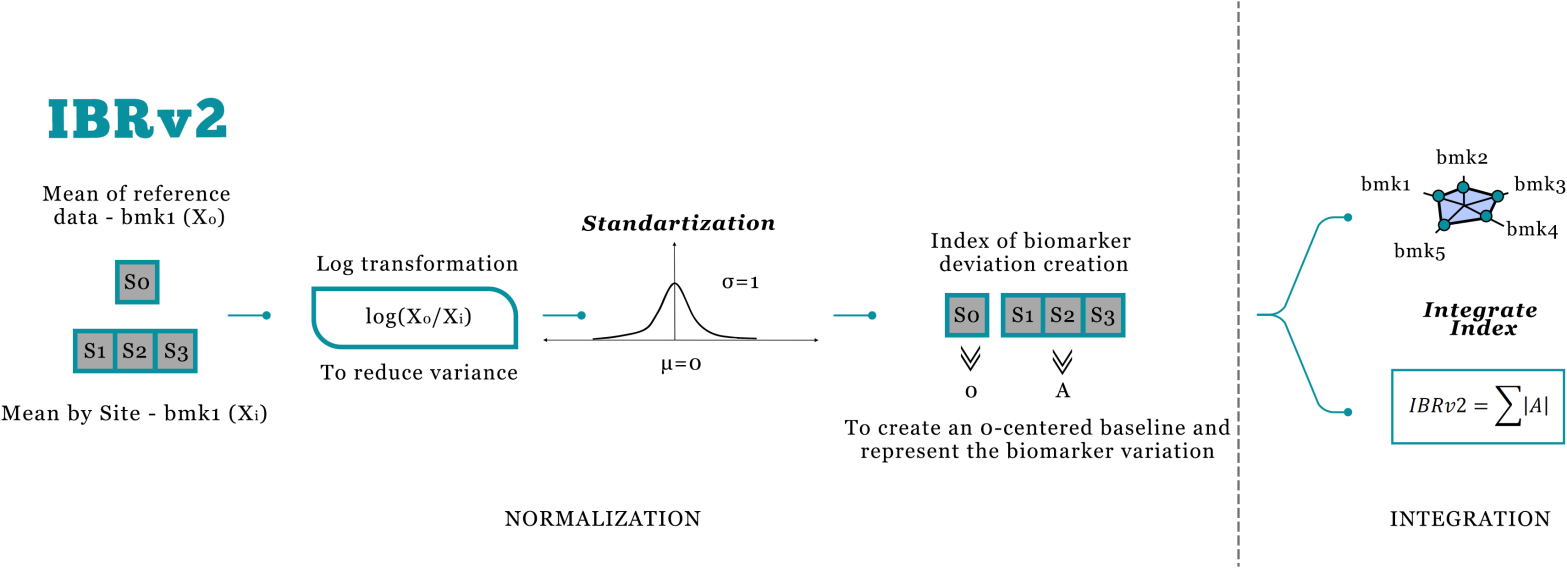
Conceptual flowchart of the integrated biomarker index version 2 calculation (IBRv2). Five hypothetical biomarkers (Bmk1, Bmk2, Bmk3, Bmk4, and Bmk5) are measured in a model organism at four different sites (S0, S2, S3, and S4, where S0 is the reference site). Continuous biomarker responses are standardized and the absolute values of the A parameters calculated for each biomarker at each investigated site are summed to obtain IBRv2.

The calculation of IBRv2 proposed by Sanchez et al. (2012) presents the steps seen in Figure 3.

**FIGURE 4:**
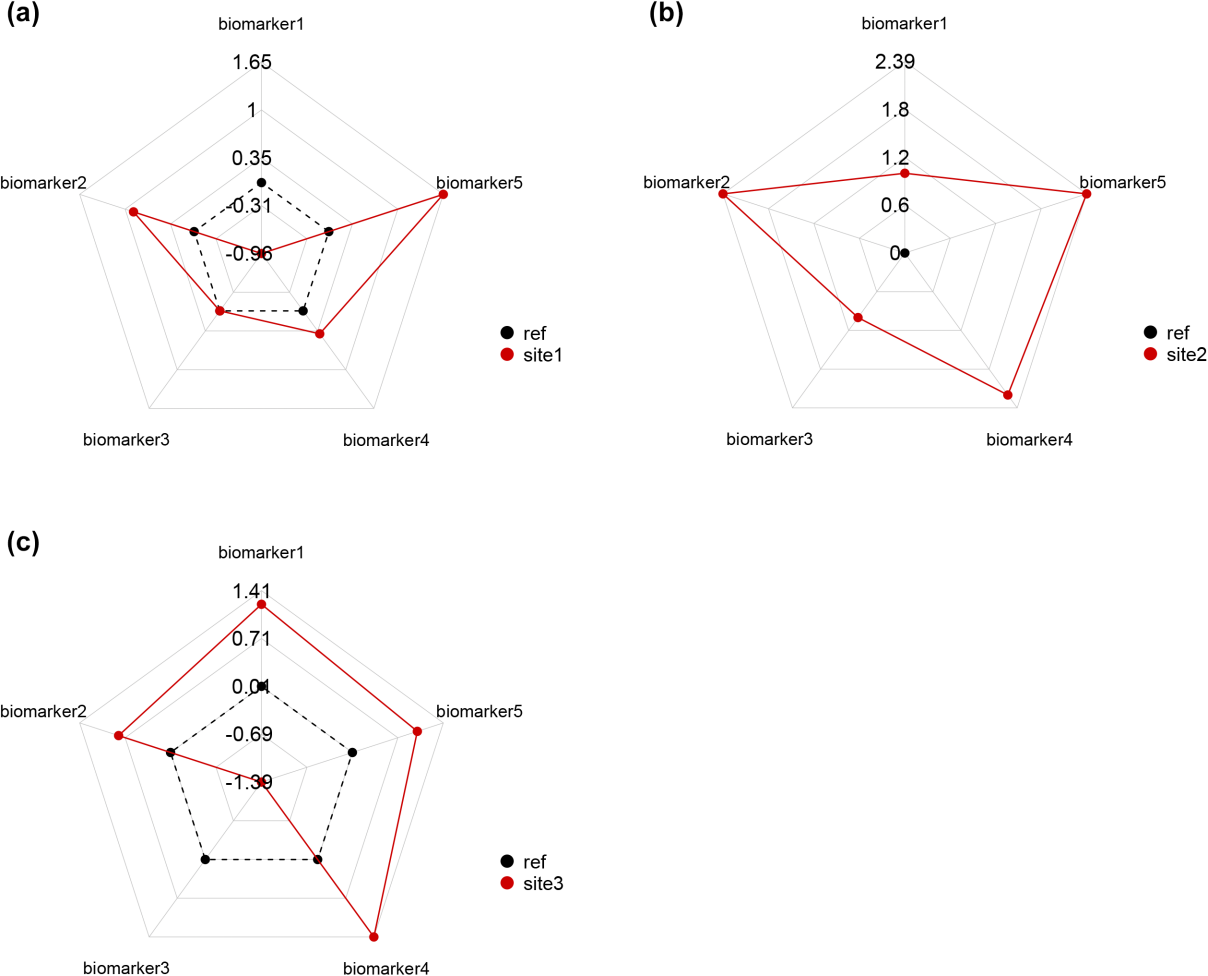
Radar chart of five biomarkers for site 1 (a), site 2 (b), and site 3 (c), obtained through the standardized values made with the *enzact2* dataset. The red dots demonstrate the up‐ and downregulation of each biomarker analyzed. The red lines demonstrate the area that corresponds to the IBRv2 value for the site in question. The black dashed lines and black points represent the reference value for each site.

Figure 4a–c shows the radar chart performed as a graphic representation of the IBRv2 index from the example dataset included in the package (*enzact2*) (Table S6). The area of the radar chart corresponds to the value of IBRv2 (generated with *ibrv2_index* function) and the value of each enzyme is their standardized form (generated with *ibrv2_bdi* function). The IBRv2 analysis will be demonstrated below. The standardized value used to generate the radar chart can be seen in Table S8.

#### 2.2.2 Example of usage



 *## install and load package*
install.packages('IBRtools')
library(IBRtools)
 *## data load*
data(enzact2)
 *## use of ibrv2_index function and save output*
ibrv2_index(enzact2) ‐> ibrv2out
 *## use of ibrv2_bdi function and save output*
ibrv2_bdi(enzact2) ‐> ibrv2bdi
 *## use of ibrv2_chart function and save output*
 *### subset output from ibrv2_bdi*
ibrv2bdi[c(1,2),] ‐> site1
ibrv2bdi[c(1,3),] ‐> site2
ibrv2bdi[c(1,4),] ‐> site3
### manual creation of axis values
site1axis=(round((seq(from=min(site1[,2:(ncol(site1))]),to=max(site1[,2:(ncol(site1))]), by=(max(site1[,2:(ncol(site1))])‐min(site1[,2:(ncol(site1))]))/4)), 2))
site2axis=(round((seq(from=min(site2[,2:(ncol(site2))]),to=max(site2[,2:(ncol(site2))]), by=(max(site2[,2:(ncol(site2))])‐min(site2[,2:(ncol(site2))]))/4)), 2))
site3axis=(round((seq(from=min(site3[,2:(ncol(site3))]),to=max(site3[,2:(ncol(site3))]), by=(max(site3[,2:(ncol(site3))])‐min(site3[,2:(ncol(site3))]))/4)), 2))
### run ibrv2_chart for each subset and save output
pdf("my_ibrv2chart.pdf")
### add manual axis values
ibrv2_chart(site1, axistype=1,seg=4, caxislabels =site1axis)
ibrv2_chart(site2, axistype=1,seg=4, caxislabels =site2axis)
ibrv2_chart(site3, axistype=1,seg=4, caxislabels =site3axis)
dev.off()




## 3 CONCLUSION

IBRtools contributes to the advancement of biomonitoring studies, expanding and facilitating the use of integrated biomarker indexes. The accuracy of these package's functions outputs was verified for accuracy with other available tools such as CalIBRi and Excel sheets, and now users are allowed to carry out analyses with the advantages of using an open‐source software, R. Currently, the package limitations are (a) we do not provide functions for the calculation of the original IBR proposed by the possibility of Beliaeff and Burgeot (2002), just for the revisited version proposed by Devin et al., 2014; (b) when using the radar chart creating functions (*ibr_chart* and *ibrv2_chart*), users might be required to modify axis values or segment quantity, which is achievable through standard R graphics commands.

## AUTHOR CONTRIBUTIONS

**Anna Resende:** Conceptualization (equal); data curation (equal); project administration (equal); software (lead); visualization (equal); writing – original draft (lead); writing – review and editing (equal). **Diego Mauro Carneiro Pereira:** Data curation (equal); formal analysis (equal); methodology (equal); software (equal); visualization (equal); writing – review and editing (equal).

## Supporting information


Table S1

## Data Availability

IBRtools is available on CRAN (https://cran.r‐project.org/package=IBRtools), Github (https://github.com/ecologicaltools/IBRtools). The datasets enzact, enzact_coef, and enzact2 are available on IBRtools package through R. The output of this package's functions can be found in Tables S1–S8. There are no other data associated with this paper.
