# Supplementary material for: IBRtools: An R package for calculating integrated biomarker indexes
Source: Ecol Evol. 2024 Feb 1;14(2):e10864. doi: 10.1002/ece3.10864 (PMC10834099; doi:10.1002/ece3.10864)
Supplement: Supplementary file 1 — Table S1 [file ECE3-14-e10864-s001.docx]

**SUPPLERMENTARY TABLES**

**TABLE S1** – Dataset with biomarker values to be used with the function *ibr_index*

| treatment | biomarker1 | biomarker2 | biomarker3 | biomarker4 | biomarker5 |
| --- | --- | --- | --- | --- | --- |
| site1 | 0.00274946 | 0.00069633 | 0.00037 | 0.75666304 | 0.0000889 |
| site1 | 0.0019533 | 0.00054422 | 0.000341 | 0.76081144 | 0.0001321 |
| site1 | 0.00271814 | 0.00059546 | 0.000351 | 0.84765915 | 0.0001796 |
| site1 | 0.00270765 | 0.00042495 | 0.01765 | 0.63785358 | 0.0001363 |
| site1 | 0.002214 | 0.00041975 | 0.00075 | 0.87421408 | 0.0000486 |
| site1 | 0.00246628 | 0.00051362 | 0.000508 | 0.76626769 | 0.0002681 |
| site1 | 0.00225564 | 0.0005073 | 0.000323 | 0.68955078 | 0.000087 |
| site1 | NA | NA | NA | NA | NA |
| site2 | 0.00225985 | 0.00020615 | 0.014507 | 0.11148184 | 0.0001103 |
| site2 | 0.00192117 | 0.00061112 | 0.000422 | 0.71302981 | 0.0001092 |
| site2 | 0.00151884 | 0.00047598 | 0.000704 | 0.58996544 | 0.0001226 |
| site2 | 0.00275916 | 0.00056148 | 0.012203 | 0.12679846 | 0.0000882 |
| site2 | 0.00176837 | 0.00039902 | 0.000354 | 0.71830183 | 0.0000745 |
| site2 | 0.00252755 | 0.0004618 | 0.000906 | 0.85878293 | 0.0001021 |
| site2 | 0.00217907 | 0.00032515 | 0.000318 | 0.17734683 | 0.0001166 |
| site2 | 0.00157868 | 0.00038761 | 0.001227 | 0.75332455 | 0.0000893 |
| site2 | 0.00206152 | 0.00054669 | 0.000588 | 0.44498099 | 0.0000897 |
| site2 | 0.00267005 | 0.00045158 |  | 0.61998664 | 0.0000822 |
| site3 | 0.00222395 | 0.00055969 | 0.008684 | 0.39649805 | 0.000128 |
| site3 | 0.00214395 | 0.00028231 | 0.000276 | 0.91681784 | 0.0001039 |
| site3 | 0.00272405 | 0.00030899 | 0.000295 | 0.60946577 | 0.0000618 |
| site3 | 0.00188876 | 0.00043765 | 0.000507 | 0.12628414 | 0.0000951 |
| site3 | 0.00272059 | 0.00052229 | 0.000526 | 0.79085668 | 0.0002801 |
| site3 | 0.00195964 | 0.00029315 | 0.000467 | 0.77234944 | 0.0000418 |
| site3 | 0.0018099 | 0.0003208 | 0.005536 | 0.6342703 | 0.000084 |
| site3 | 0.00212396 | 0.00037607 | 0.000231 | 0.66315815 | 0.0001248 |
| site3 | 0.00278725 | 0.00031785 | 0.000458 | 0.34690789 | 0.0001075 |
| site3 | 0.00172009 | NA | 0.000202 | 0.78563329 | NA |

**TABLE S2** – Dataset with coefficient values to be used with the function *ibr_index*

| treatment | biomarker1 | biomarker2 | biomarker3 | biomarker4 | biomarker5 |
| --- | --- | --- | --- | --- | --- |
| site1 | 1 | -1 | 1 | -1 | 1 |

**TABLE S3** – Dataset with output values from the first dataframe of the returned list from the function *ibr_index*

| treatment | value |
| --- | --- |
| site1 | 1.83529927 |
| site1 | 1.25359805 |
| site1 | 3.08889732 |
| site1 | 2.46983663 |
| site1 | 1.25359805 |
| site1 | 1.21623857 |
| site1 | 3.05153784 |
| site1 | 1.21623857 |
| site1 | 3.05153784 |
| site1 | 1.21623857 |
| site1 | 2.46983663 |
| site1 | 2.46983663 |
| site1 | 3.08889732 |
| site1 | 2.46983663 |
| site1 | 1.83529927 |
| site1 | 1.25359805 |
| site1 | 1.21623857 |
| site1 | 1.25359805 |
| site1 | 1.83529927 |
| site1 | 3.05153784 |
| site1 | 3.08889732 |
| site1 | 3.08889732 |
| site1 | 3.05153784 |
| site1 | 1.83529927 |
| site2 | 2.91855622 |
| site2 | 1.07421906 |
| site2 | 2.9280188 |
| site2 | 1.08368165 |
| site2 | 1.84433716 |
| site2 | 1.84433716 |
| site2 | 2.15790071 |
| site2 | 1.07421906 |
| site2 | 2.9280188 |
| site2 | 1.84433716 |
| site2 | 1.08368165 |
| site2 | 1.08368165 |
| site2 | 2.15790071 |
| site2 | 1.08368165 |
| site2 | 2.91855622 |
| site2 | 1.84433716 |
| site2 | 1.07421906 |
| site2 | 1.07421906 |
| site2 | 2.91855622 |
| site2 | 2.9280188 |
| site2 | 2.15790071 |
| site2 | 2.9280188 |
| site2 | 2.15790071 |
| site2 | 2.91855622 |
| site3 | 1.2351049 |
| site3 | 1.32982822 |
| site3 | 1.89987998 |
| site3 | 2.19540165 |
| site3 | 1.63845262 |
| site3 | 1.83925097 |
| site3 | 1.91252153 |
| site3 | 1.65109417 |
| site3 | 2.22114593 |
| site3 | 1.83925097 |
| site3 | 2.31586925 |
| site3 | 2.19540165 |
| site3 | 1.71172318 |
| site3 | 2.31586925 |
| site3 | 1.3555725 |
| site3 | 1.63845262 |
| site3 | 1.65109417 |
| site3 | 1.32982822 |
| site3 | 1.3555725 |
| site3 | 2.22114593 |
| site3 | 1.71172318 |
| site3 | 1.89987998 |
| site3 | 1.91252153 |
| site3 | 1.2351049 |

**TABLE S4** – Dataset with output values from the second dataframe of the returned list from the function *ibr_index*

| treatment | ibr_mean | ibr_sd |
| --- | --- | --- |
| site1 | 2.15256795 | 0.78806731 |
| site2 | 2.00111893 | 0.77468985 |
| site3 | 1.77548708 | 0.3502225 |

**TABLE S5** – Dataset with output values from the function *ibr_std* when using coefficients from *enzact_coef* dataset

| group | biomarker1 | biomarker2 | biomarker3 | biomarker4 | biomarker5 |
| --- | --- | --- | --- | --- | --- |
| site1 | 1.9350648 | 0 | 1.32174273 | 0 | 1.99450463 |
| site2 | 0 | 1.15209678 | 1.9607741 | 1.97804617 | 0 |
| site3 | 0.52976172 | 1.99185373 | 0 | 1.24495279 | 0.86894226 |

**TABLE S6** – Dataset with biomarker values to be used with the function *ibrv2_index*

| sites | biomarker1 | biomarker2 | biomarker3 | biomarker4 | biomarker5 |
| --- | --- | --- | --- | --- | --- |
| ref | 0.00367946 | 0.00140057 | 0.15255104 | 0.14231642 | 0.00128077 |
| ref | 0.0065889 | 0.00111225 | 0.10991809 | 0.25675552 | 0.00109341 |
| ref | 0.00727577 | 0.00111714 | 0.12416306 | 0.23505584 | 0.00097551 |
| ref | 0.0062443 | 0.00105929 | 0.13343544 | 0.24963614 | 0.0006305 |
| ref | 0.00355339 | 0.00126038 | 0.1964501 | 0.22310303 | 0.00086865 |
| ref | 0.00411535 | 0.00064974 | 0.12052274 | 0.28056515 | 0.00074124 |
| ref | 0.00378027 | 0.00094989 | 0.14803214 | 0.2777104 | 0.00103673 |
| ref | 0.00370673 | 0.00100039 | 0.27549425 | 0.28521456 | 0.00109429 |
| ref | 0.00433899 | 0.00052197 | 0.11843582 | 0.19217144 | 0.00062403 |
| ref | 0.00307636 | 0.00126154 | 0.12185148 | 0.20249087 | 0.00095768 |
| site1 | 0.0045169 | 0.00069757 | 0.09905447 | 0.30217989 | 0.00084811 |
| site1 | 0.00393673 | 0.000916 | 0.19531238 | 0.24846371 | 0.00122212 |
| site1 | 0.00319508 | 0.00078406 | 0.09345947 | 0.09351594 | 0.00072867 |
| site1 | 0.00325595 | 0.00095094 | 0.13253931 | 0.24659194 | 0.00149726 |
| site1 | 0.00254842 | 0.00153698 | 0.15393391 | 0.29051114 | 0.00161401 |
| site1 | 0.00344686 | 0.00115892 | 0.18379374 | 0.24629423 | 0.00126119 |
| site1 | 0.0040255 | 0.00154775 | 0.16440451 | 0.24240641 | 0.00138209 |
| site1 | 0.00448188 | 0.00164229 | 0.19365482 | 0.26977565 | NA |
| site1 | 0.00476405 | 0.00109394 | 0.13863345 | 0.29856786 | 0.00114455 |
| site2 | 0.00549357 | 0.00218593 | 0.17304929 | 0.37217438 | 0.00178396 |
| site2 | 0.00395884 | 0.00147255 | 0.22070913 | 0.36666194 | 0.00104573 |
| site2 | 0.00719761 | 0.00142446 | 0.1816104 | 0.40807583 | 0.00134732 |
| site2 | 0.00517638 | 0.00080127 | 0.121058 | 0.23952288 | 0.00078772 |
| site2 | 0.00512232 | 0.00086795 | 0.05875069 | 0.21764284 | 0.0008745 |
| site2 | 0.00537289 | 0.00110099 | 0.15357766 | 0.25475663 | 0.0010486 |
| site2 | 0.00484073 | 0.00165188 | 0.21666322 | 0.33744794 | 0.0016556 |
| site2 | 0.00727258 | 0.00208994 | 0.158675 | 0.29979609 | 0.00197597 |
| site2 | 0.00629235 | 0.0010993 | 0.21208482 | 0.33265459 | 0.00193763 |
| site2 | 0.00642465 | 0.00104976 | 0.12023289 | 0.31322783 | 0.00112484 |
| site3 | 0.00538467 | 0.00099441 | 0.12748962 | 0.27224599 | 0.00094302 |
| site3 | 0.00892244 | 0.0011028 | 0.13468313 | 0.27870661 | 0.00145216 |
| site3 | 0.0056816 | NA | 0.11320313 | 0.2694979 | 0.00092538 |
| site3 | 0.00758037 | 0.00167859 | 0.12888754 | 0.2272737 | 0.00135133 |
| site3 | 0.00538973 | 0.00052223 | 0.06277263 | 0.23979127 | 0.00115552 |
| site3 | 0.00764736 | 0.00110132 | 0.15814969 | 0.28132221 | 0.00090522 |
| site3 | 0.00288239 | 0.00125607 | 0.19947257 | 0.28477187 | NA |
| site3 | 0.00296183 | 0.00105319 | 0.14779144 | 0.31904853 | NA |
| site3 | 0.00719531 | 0.0013743 | 0.15132669 | 0.36457795 | 0.00095327 |

**TABLE S7** – Dataset with output values from the function *ibrv2_index*

| group | indexvalue |
| --- | --- |
| ref | 0 |
| site1 | 3.98236897 |
| site2 | 9.04120942 |
| site3 | 5.83029601 |

**TABLE S8** – Dataset with output values from the function *ibrv2_bdi*

| group | biomarker1 | biomarker2 | biomarker3 | biomarker4 | biomarker5 |
| --- | --- | --- | --- | --- | --- |
| ref | 0 | 0 | 0 | 0 | 0 |
| site1 | -0.95989527 | 0.88037593 | 0.04180005 | 0.44969894 | 1.65059878 |
| site2 | 1.00607885 | 2.39384029 | 1.0440425 | 2.23794445 | 2.35930334 |
| site3 | 1.20821605 | 0.79037568 | -1.38971684 | 1.40863124 | 1.03335619 |
